# Reliability of Childhood Autism Rating Scale, Second Edition, Assessments Using Telepsychiatry for Children With Neurodevelopmental Disorders and Their Caregivers: Randomized Feasibility Study

**DOI:** 10.2196/82784

**Published:** 2026-10-06

**Authors:** Shunya Kurokawa, Kensuke Nomura, Nana Hosogane, Takashi Nagasawa, Yuko Kawade, Yu Matsumoto, Shuichi Morinaga, Yuriko Kaise, Ayana Higuchi, Akiko Goto, Naoko Inada, Masaki Kodaira, Taishiro Kishimoto

**Affiliations:** 1 Center for the Promotion of Interdisciplinary Research in Medicine and Life Science, Keio University School of Medicine, Mori JP Tower F7, 1-3-1, Azabudai, Minato-ku, Tokyo, Japan, 81 3-5363-3219; 2 Department of Child Psychiatry, Shimada Ryoiku Medical Center for Challenged ChildrenTokyo, Japan; 3 Department of Neuropsychiatry, School of Medicine, Keio University, Tokyo, Japan; 4 Department of Child and Adolescent Mental Health, Aiiku Clinic, Tokyo, Japan; 5 Department of Child and Adolescent Psychiatry, Tokyo Metropolitan Children's Medical Center, Tokyo, Japan; 6 Tsurugaoka Garden Hospital, Tokyo, Japan; 7 Hiratsuka City Hospital, Kanagawa, Japan; 8 Department of Clinical Psychology, Taisho University, Tokyo, Japan

**Keywords:** attention-deficit/hyperactivity disorder, autism spectrum disorder, telepsychiatry, online assessment, Childhood Autism Rating Scale

## Abstract

**Background:**

The COVID-19 pandemic has increased the demand for telepsychiatry in neurodevelopmental assessment; however, few studies have examined the interrater reliability of clinician-rated autism scales, such as the Childhood Autism Rating Scale, Second Edition (CARS2), when used remotely. Although the feasibility and validity of telehealth assessments have been explored previously, the consistency between remote and in person CARS2 ratings by different clinicians remains underinvestigated.

**Objective:**

The objective of this study was to evaluate agreement between CARS2 total scores obtained by different evaluators using telepsychiatry and face-to-face assessments in children with autism spectrum disorder (ASD) and/or attention-deficit/hyperactivity disorder (ADHD).

**Methods:**

In this randomized feasibility study, 75 children aged 6 to 17 years with *DSM-5 (Diagnostic and Statistical Manual of Mental Disorders [Fifth Edition])* diagnoses of ASD, ADHD, or both were enrolled at 5 medical institutions in Japan; 73 of 75 were included in the final analysis. Each participant underwent 2 independent CARS2 evaluations, one face-to-face and the other via telepsychiatry, conducted by different evaluators who were blinded to each other’s ratings. Remote assessments were conducted using a secure video platform with caregiver facilitation. Agreement was assessed using intraclass correlation coefficients (ICCs) for the overall sample and subgroups defined by primary diagnosis, sex, and age.

**Results:**

The overall ICC for CARS2 total scores between face-to-face and telepsychiatry assessments was 0.582 (95% CI 0.407‐0.717), indicating moderate agreement. The ICC was 0.546 among participants with ASD as the primary diagnosis and 0.495 among those with ADHD as the primary diagnosis. Point estimates were higher among girls than boys (0.666 vs 0.495) and among children aged 11 years or older than among those aged 10 years or younger (0.676 vs 0.419). In a sensitivity analysis including all participants with an ASD diagnosis, the ICC was 0.580.

**Conclusions:**

CARS2 total scores obtained through telepsychiatry showed moderate agreement with scores obtained through face-to-face assessment when the assessments were conducted by different evaluators in different settings. Higher point estimates were observed among older children and girls, although these subgroup findings should be interpreted cautiously. Telepsychiatry-based CARS2 assessment may be considered a complementary approach rather than a replacement for comprehensive face-to-face evaluation. Larger studies using more standardized assessment protocols are needed.

## Introduction

Autism spectrum disorder (ASD) is a neurodevelopmental condition characterized by persistent deficits in social communication and interaction alongside restricted, repetitive patterns of behavior [1]. The global prevalence of ASD is estimated to be approximately 1% to 2% of the population, though reported rates vary by region and have generally increased in recent decades [2]. Accurate diagnosis and assessment of ASD severity typically requires a combination of direct behavioral observations of the child and in-depth caregiver interviews.

The COVID-19 pandemic has accelerated the adoption of telepsychiatry for ASD evaluation, prompting extensive research on the reliability and validity of remote diagnostic tools. Early studies and reviews have generally been encouraging; telepsychiatric autism evaluations show a high agreement with in person diagnoses and have been well received by families and clinicians [3,4]. These findings suggest that when appropriately adapted, remote ASD assessment methods can feasibly bridge gaps in access without compromising diagnostic accuracy.

Structured caregiver interviews, such as the Autism Diagnostic Interview-Revised (ADI-R) or the Diagnostic Interview for Social and Communication Disorders, are particularly amenable to remote use because they rely on verbal reports rather than live observations of the child. Because these interviews rely on parent-reported histories rather than on tasks administered directly to the child, their core content can be preserved over video.

For example, telepsychiatric administration of the ADI-R has shown strong agreement with in person evaluations. A US clinical trial of 51 toddlers (aged 18‐36 months) found that a caregiver-mediated teleassessment achieved 86% agreement with standard face-to-face ADI-R ASD diagnoses [5]. Notably, a randomized study likewise reported no differences in diagnostic consistency between in person and video-based ADI-R assessments; interrater reliability on ADI-R scores was statistically equivalent across modes [6]. These findings indicate that the ADI-R diagnostic algorithm remains robust when interviews are conducted using video.

In contrast, adapting direct behavioral observation tools, such as the Autism Diagnostic Observation Schedule (ADOS), for telehealth has been more challenging. The ADOS is a semistructured play-based assessment often regarded as a gold standard for ASD diagnosis, but it traditionally requires face-to-face interaction with the child. In one study, when clinicians attempted to administer the ADOS over video, they could achieve approximately 86% concordance in overall diagnostic decisions compared with in person evaluations. However, item-level reliability was markedly lower for remote behavioral observations: the caregiver interview portion (ADI-R) maintained substantial interrater agreement (κ=0.82) via telehealth, whereas the remote ADOS items showed only moderate agreement (κ=0.5) [6].

However, certain telehealth adaptations of the ADOS have yielded promising results under specific conditions. For instance, a modified remote administration of the ADOS for verbally fluent adults demonstrated an intraclass correlation coefficient (ICC) of approximately 0.92 for diagnostic classification, comparable to an in person evaluation [7].

These mixed outcomes illustrate that while remote behavioral observations are feasible, ensuring high reliability for all components of an autism assessment battery can be difficult when conducted online.

Beyond full diagnostic interviews and observational batteries, more streamlined ASD rating scales have been explored for remote assessment. The Childhood Autism Rating Scale, Second Edition (CARS2), is one such tool that has attracted interest in the telehealth context. The CARS2 is a clinician-rated scale that quantifies ASD symptom severity across 15 domains and is valued for its brief administration time and minimal training requirements [8]. Traditionally, CARS2 scoring integrates information from multiple sources (caregiver reports, clinical records, and direct observation); however, it can also be based on focused direct observation of the child alone.

During the pandemic, this instrument was adapted for remote use by having clinicians observe and interact with children via videoconference, an approach often termed the remote CARS2 (rCARS2) assessment. A recent validation study of the rCARS2 against the in person ADOS, Second Edition (ADOS-2), reported promising results: remote CARS2 scores were strongly correlated with ADOS-2 scores (Spearman ρ=0.64) [9]. These findings support the utility of CARS2 as an efficient telehealth-compatible observation tool for ASD, capable of maintaining good concordance with gold-standard in person assessments.

Despite a growing body of literature on telehealth-based ASD assessments, a notable gap remains in our understanding of the interrater reliability of these remote assessment tools. To date, most tele-ASD studies have focused on validity (ie, agreement with in person diagnoses or standard instruments) and feasibility. Far fewer have examined whether different clinicians observing the same child, either in person or via video, assign consistent ratings. Interrater reliability is a critical property of any diagnostic instrument that reflects consistency and objectivity across examiners. Notably, we are unaware of any prior work that has specifically evaluated the interrater reliability of the CARS2 when administered via telepsychiatry.

However, prior research suggests that clinician-rated scales of attention-deficit/hyperactivity disorder (ADHD) can be implemented reliably via telehealth, with substantial agreement between telepsychiatry and face-to-face assessments [10]. Building on this foundation, the present study aimed to evaluate the interrater reliability of the CARS2 administered via telepsychiatry versus in person. Clarifying the reliability of remote CARS2 assessments is clinically important for establishing trusted telehealth practices in ASD diagnosis and ensuring that evaluations yield consistent results regardless of whether they occur face-to-face or online.

## Methods

### Participants

Patients were recruited from Keio University Hospital and 4 collaborating institutions (Shimada Ryoiku Medical Center for Challenged Children, Aiiku Clinic, Tokyo Metropolitan Children’s Medical Center, and Tsurugaoka Garden Hospital). The inclusion criteria were as follows: (1) a confirmed *DSM-5 (Diagnostic and Statistical Manual of Mental Disorders [Fifth Edition])* diagnosis of ASD, ADHD, or both, (2) aged between 6 and 17 years at the time of obtaining consent, and (3) stable pharmacotherapy, if applicable, for at least 3 months before consent.

Exclusion criteria included (1) hearing or visual impairment in either the child or caregiver that interfered with evaluation despite corrective devices, (2) absence of caregivers who are knowledgeable about the participant’s early developmental history, (3) presence of severe comorbid psychiatric symptoms (eg, hallucinations and delusions) as determined by clinicians, and (4) initiation of new treatments, such as pharmacotherapy or psychotherapy, anticipated during the study period. The study protocol was approved by the Ethics Committee of Keio University School of Medicine (20190301). This study was registered in the UMIN Clinical Trial Registry (UMIN000039860).

### Baseline Assessments

Demographic and clinical data, including age, sex, diagnosis, medication history, illness duration, and intelligence test results, were extracted from medical records. Baseline assessment included the ADHD Rating Scale-IV (ADHD-RS-IV), which was rated by a trained clinician through a brief semistructured interview with the caregiver; the standard 18 items (inattention, 9; hyperactivity/impulsivity, 9) were scored. The Conners 3 parent form was completed by caregivers and scored according to the test manual. The Autism-Spectrum Quotient–Japanese version for children, the Strengths and Difficulties Questionnaire to evaluate social adjustment, and the Short Sensory Profile were used to assess sensory characteristics.

### Study Procedure

The CARS2 was used as the primary measure to evaluate agreement between face-to-face and telepsychiatry assessments conducted by different evaluators. The CARS2 includes 2 versions, the Standard Version (CARS2-ST) and the High-Functioning Version (CARS2-HF), with the latter recommended for children aged 6 years and older who have an IQ greater than 80 or relatively intact verbal abilities. In designing this study, we assumed patients without comorbid intellectual disability.

Following the baseline assessment, each participant underwent 2 independent CARS2 evaluations conducted by different evaluators: 1 face-to-face assessment and 1 telepsychiatry assessment. Participants were randomly assigned to 1 of 2 assessment sequences: face-to-face assessment followed by telepsychiatry assessment or telepsychiatry assessment followed by face-to-face assessment. The randomization sequence was generated by the study coordinator at each participating site using a random number generator. To minimize recall and order effects, the interval between the 2 evaluations ranged from 2 weeks to 3 months. Each evaluator was blinded to the other evaluator’s ratings.

The evaluators were licensed psychologists trained in developmental assessment. Prior to this study, evaluators underwent standardized training and demonstrated adequate interrater reliability in 5 pilot cases, confirming the consistency and accuracy of the CARS2 tool.

In this study, we set structured prompts to help examiners avoid missing key behaviors and to minimize the effects of different raters. To minimize modality-specific bias while sampling a broad range of social communication behaviors, we embedded 3 structured observation blocks—Uno play, semistructured social interviews, and reverse interviews using photo prompts— along with the standard CARS2 Questionnaire for Parents or Caregivers.

Uno play with a caregiver elicited spontaneous turn taking, joint attention, gesture use, and affect, allowing direct scoring of the following CARS2 items: 1, Relating to People; 2, Imitations; 3, Emotional Responses; 7, Visual Responses; 12, Nonverbal Communication; and 13 Activity Levels.

The semistructured social interview followed a fixed sequence of anchor questions (hobbies, chores, school strengths/weaknesses, family pets, and sources of happiness, sadness, fear, anger, and loneliness) with body-state probes, such as “How does your body feel when you are happy?” These standardized topic shifts left room for natural dialogue, providing comparable material for item 3, Emotional Response; item 6, Adaptation to Change; item 10, Fear or Nervousness; item 11, Verbal Communication; item 12, Nonverbal Communication; and item 14, Level/Consistency of Intellectual Response.

Next, the reverse interview asked the child to “interview the examiner” for two minutes using staged photographs (birthday party and borrowing an umbrella) as optional cues. This segment sampled spontaneous question generation, perspective taking, and imaginative language, informing items 1, 11, 12, and 14, and the overall item 15, General Impressions. Screen sharing ensured identical visual stimuli in the telepsychiatric condition.

The remote smartphone assessment tool Curon (MICIN Co Ltd) was used for the telemedicine evaluation. The participants were seated in front of a smartphone in their homes and introduced to a remote evaluator. Remote evaluators administered their evaluations in a room at the Keio University Hospital using a personal computer. Although it was assumed that a download and upload environment of 50 Mbps or higher would be ideal for remote video applications, stable communication was achieved at approximately 5 Mbps, which in most cases met the standards of the Japanese standard 4G network. Assessments began after confirming that there were no interruptions in the video or audio environment.

### Statistical Analysis

Interrater reliability was assessed using ICCs for continuous CARS2 scores, categorized according to standard performance parameters: almost perfect (0.81‐1), substantial (0.61‐0.80), moderate (0.41‐0.60), fair (0.21‐0.40), and slight (0‐0.20) [11]. Interrater agreement between face-to-face and telepsychiatry ratings was estimated with a standard ICC suitable for paired ratings from 2 independent evaluators; intrarater reliability was not assessed by design. A sample size of at least 31 participants was calculated to achieve an 80% power, assuming an ICC of 0.8 and aiming for a 95% CI width of 0.3. Normally distributed data are reported as mean (SD), and categorical variables are reported as numbers (%). Statistical analyses were performed using SPSS software (version 25.0, IBM Corp). In a post hoc sensitivity analysis, we reestimated ICCs after excluding ADHD-only participants (ie, restricting to children with an ASD diagnosis present) to assess whether diagnostic heterogeneity materially affected reliability.

### Ethical Considerations

The ethics committee of Keio University School of Medicine approved the study protocol (approval number 20190301). This study was registered in the UMIN Clinical Trial Registry (UMIN000039860). Written informed consent was obtained from the caregivers, and age-appropriate informed assent was obtained from the participating children. Data confidentiality was maintained through anonymization, and secure storage was ensured at all times.

## Results

### Participants

Data were collected from August 2020 to February 2022. The demographic and clinical characteristics of the participants are presented in Table 1, and a flowchart of the study procedure is shown in Figure 1. Of the 75 participants who provided informed consent, 1 did not complete the second assessment because of time limitations. One additional participant with a moderate intellectual disability completed the assessments using the CARS2-ST; because this was the only participant assessed using this version, the participant was excluded from the final analysis. Thus, 73 participants were included in the final analysis.

**Table 1.** Demographic and clinical characteristics of the 73 children included in the final analysis of a randomized feasibility study comparing face-to-face and telepsychiatry Childhood Autism Rating Scale, Second Edition (CARS2), assessments at 5 medical institutions in Japan, August 2020 to February 2022.

Variable	Participants (N=73)	Participants with a primary ADHD^a^ diagnosis (N=43)	Participants with a primary ASD^b^ diagnosis (N=30)	Participants with comorbid ASD and ADHD (N=22)
Female sex, n (%)	17 (23.3)	12 (27.9)	5 (16.7)	7 (31.8)
Age (years)	10.4 (2.5)	10.5 (2.4)	10.2 (2.6)	10.2 (2.5)
Mean SDQ^c^ (SD)	22.06 (5.25)	21.09 (5.35)	23.24 (4.96)	23.30 (5.22)
Mean AQ^d^ (SD)	24.56 (7.37)	22.86 (6.72)	27.03 (7.68)	26.94 (7.80)
Mean Conners 3 score (SD)	112.70 (38.49)	108.34 (39.94)	118.41 (36.40)	119.41 (36.67)
Mean SSP^e^ score (SD)	70.90 (20.37)	67.44 (20.38)	75.63 (19.73)	76.0 (19.19)
Mean CARS2 (face-to-face) score (SD)	35.88 (4.88)	34.19 (4.49)	38.25 (4.45)	37.86 (4.55)
Mean ADHD-RS-IV^f^ total score (face-to-face) (SD)	27.14 (9.91)	26.88 (10.38)	27.52 (9.35)	27.88 (9.15)
Mean ADHD-RS-IV hyperactivity/impulsiveness score (face-to-face) (SD)	7.99 (6.07)	7.95 (6.60)	8.03 (5.33)	8.34 (5.25)
Mean ADHD-RS-IV total inattention score (face-to-face) (SD)	19.15 (5.32)	18.93 (5.28)	19.48 (5.46)	19.53 (5.27)

a ADHD: attention-deficit/hyperactivity disorder.

b ASD: autism spectrum disorder.

c SDQ: Strengths and Difficulties Questionnaire.

d AQ: autism spectrum quotient.

e SSP: Short Sensory Profile.

f ADHD-RS-IV: Attention-Deficit/Hyperactivity Disorder Rating Scale-IV.

**Figure 1.**
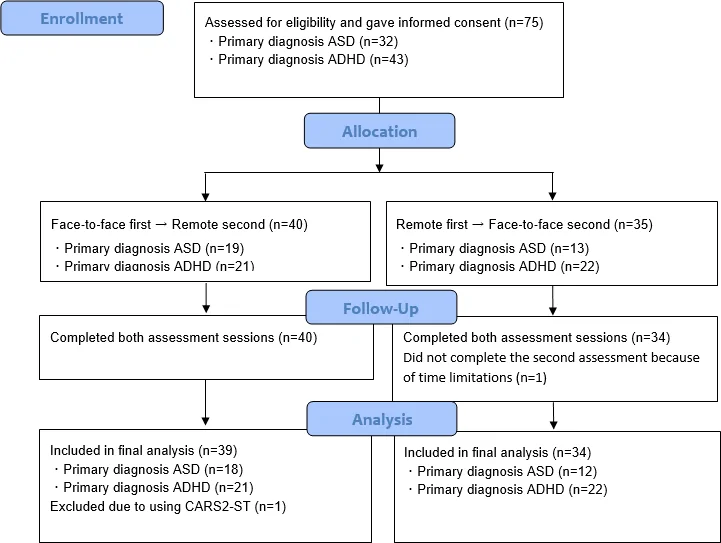
CONSORT (Consolidated Standards of Reporting Trials) diagram of participant flow through the study. ADHD: attention-deficit/hyperactivity disorder; ASD: autism spectrum disorder; CARS2-ST: Childhood Autism Rating Scale, Second Edition—Standard Version.

Of the 73 participants, 2 were recruited from Keio University Hospital, 42 from Shimada Ryoiku Medical Center for Challenged Children, 13 from Aiiku Clinic, 10 from Tokyo Metropolitan Children’s Medical Center, and 6 from Tsurugaoka Garden Hospital. Seventeen participants (23.3%) were girls and 56 (76.7%) were boys. All participants were Japanese. Their ages ranged from 6 to 16 years, with a mean age of 10.4 (SD 2.5) years.

The primary diagnosis was ASD in 30 participants (41.1%) and ADHD in 43 participants (58.9%). Regarding diagnostic status, 30 participants (41.1%) had ASD only, 21 (28.8%) had ADHD only, and 22 (30.1%) had both ASD and ADHD. All 22 participants with comorbid ASD and ADHD had ADHD designated as their primary diagnosis.

Among the 61 caregivers for whom information on previous remote video call use was available, 47 (77%) had previous experience with remote video calls and 14 (23%) did not. The median interval between the 2 assessments was 29 days (IQR 22‐37; range 14‐90 days).

In Table 1, data are presented as mean (SD) unless otherwise indicated. The primary diagnosis groups are mutually exclusive and sum to the full sample of 73 participants. The comorbid ASD and ADHD column represents an overlapping subgroup; all 22 participants in this subgroup had ADHD designated as their primary diagnosis.

### Agreement Between Face-to-Face and Telepsychiatry CARS2 Assessments

The ICC obtained during preliminary evaluator training was 0.885 (95% CI 0.418‐0.987; *P*=.005). The relationship between face-to-face and telepsychiatry CARS2 total scores is shown in Figure 2. In the overall sample, the ICC was 0.582 (95% CI 0.407‐0.717; *P*<.001), which fell within the prespecified moderate range. Among participants with ADHD as the primary diagnosis, the ICC was 0.495 (95% CI 0.228‐0.693; *P*<.001), indicating moderate agreement. Among participants with ASD as the primary diagnosis, the ICC was 0.546 (95% CI 0.238‐0.755; *P*<.001), also indicating moderate agreement. In the post hoc sensitivity analysis excluding the 21 ADHD-only participants and therefore including the 52 participants with an ASD diagnosis, the ICC was 0.580 (95% CI 0.301‐0.768; *P*<.001). This estimate was similar to the overall ICC of 0.582. The ICC was 0.495 (95% CI 0.228‐0.693; *P*<.001) among boys and 0.666 (95% CI 0.287‐0.864; *P*=.001) among girls. Among participants aged 11 years or older (n=37), the ICC was 0.676 (95% CI 0.433‐0.828; *P*<.001), whereas among those aged 10 years or younger (n=36), the ICC was 0.419 (95% CI 0.127‐0.644; *P*=.003).

**Figure 2.**
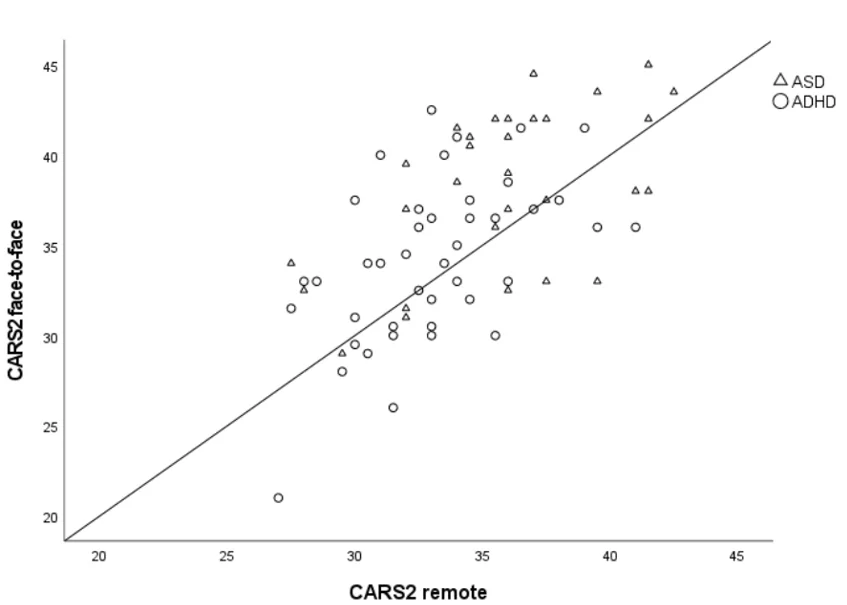
Relationship between telepsychiatry and face-to-face Childhood Autism Rating Scale, Second Edition (CARS2), total scores among 73 children aged 6 to 16 years with autism spectrum disorder (ASD) and/or attention-deficit/hyperactivity disorder (ADHD) recruited from 5 medical institutions in Japan between August 2020 and February 2022. Each point represents 1 participant; triangles indicate ASD as the primary diagnosis, while circles indicate ADHD as the primary diagnosis. The solid diagonal line represents the line of identity (y=x).

## Discussion

### Principal Results

This study aimed to evaluate agreement between CARS2 total scores obtained by different evaluators using face-to-face and telepsychiatry assessments in children with ASD and/or ADHD. The overall agreement was moderate, with an ICC of 0.582. Moderate agreement was also observed both among participants with ASD as the primary diagnosis (ICC 0.546) and among those with ADHD as the primary diagnosis (ICC 0.495). The sensitivity analysis including the 52 participants with an ASD diagnosis yielded an ICC of 0.580, which was similar to the estimate for the overall sample. This finding suggests that inclusion of participants with ADHD only did not substantially alter the overall estimate. Point estimates were higher among girls than boys and among participants aged 11 years or older than among younger participants. However, these subgroup analyses were exploratory, the CIs were wide, and no formal statistical comparisons between subgroup ICCs were performed. Therefore, these differences should not be interpreted as establishing sex- or age-related effects. Overall, the findings indicate that face-to-face and telepsychiatry CARS2 assessments captured overlapping information. However, because different evaluators conducted the assessments in different settings, the findings do not establish equivalence or interchangeability between the 2 assessment approaches.

### Comparison With Prior Work

Our findings align with and add to earlier research on telepsychiatric testing for autism. The moderate match we saw between the remote and in person CARS2 scores was almost the same size as that reported by other teams. For instance, Reese et al [6] found that a video caregiver interview (using the ADI-R) kept very high agreement (κ=0.82), but the observational ADOS part fell to only moderate (κ=0.50) when performed remotely. A newer “brief” protocol that pairs the Brief Observation of Symptoms of Autism with a parent interview reached κ=0.66 [12]. In contrast, adults tested using 2 cameras and an operator who could zoom and pan showed excellent agreement (ICC=0.92) [7]. Together, these studies suggest that caregiver interview components may retain higher agreement during telehealth assessments than direct behavioral observation components. More standardized camera positioning and observation procedures may help reduce modality-related variation, although these approaches require further evaluation.

The location where the test occurs is also important. Telepsychiatry allows clinicians to observe children in their everyday spaces, which can reveal their natural behavior. However, small apartments, background noise, and parents juggling the camera made examinations less uniform. We found lower agreement in younger children, mirroring our earlier work with telepsychiatry ADHD Rating Scale ratings [10]. Some children were quieter or more restrained on video, a point that matches reports that autism traits can shift with context [13]. Our data quantify how these shifts reduce scoring consistency, especially in crowded urban Japanese homes where private spaces are scarce. We also noted that parents sitting next to their child for the entire call sometimes hesitated to discuss problem behaviors openly, which could subtly change certain CARS2 item scores.

Technology adds another layer. Eye contact, facial expressions, and small hand gestures are tricky to judge through a single webcam. Two-camera systems or remote-controlled units provide a clearer view but are difficult to roll out in everyday care [7,13]. However, the effects of age and sex are poorly understood. We observed higher agreement in older children, who stayed on task better, and in girls. One possible explanation is that the girls in our sample had more obvious symptoms, because girls are often diagnosed only when difficulties are very clear despite “camouflaging” [14]. The higher point estimate among girls should be interpreted cautiously because the female subgroup was small and the CI was wide. The present study was not designed to determine the mechanisms underlying possible sex-related differences in agreement. Future studies should test whether this finding holds true for larger sample sizes.

Finally, our choice of the CARS2 is a part of the picture. During COVID-19, many clinics switched to the remote CARS2 because gold-standard ADOS visits were impossible [15]. Bertollo’s [9] dissertation showed a good match between the remote CARS2 and in person ADOS (ρ=0.64 and 83% diagnostic accuracy). What was still missing was a clear look at how well two different clinicians agree when they rate the same child in the clinic and by telepsychiatry, which is exactly what our study provides. The remaining gaps that we found point to straightforward upgrades: clearer camera guidelines, structured parent coaching, extrashort tasks to prompt key behaviors, and, possibly, low-cost secondary cameras. These approaches may improve the standardization of remote CARS2 assessment, but their effects on agreement should be evaluated prospectively.

### Limitations

Some limitations of our study should be acknowledged when interpreting the findings. First, our sample was modest and drawn exclusively from Japanese tertiary clinics treating children already diagnosed with ASD and/or ADHD, which may limit the generalizability to undiagnosed groups or different cultural settings. Also, as the present study was designed around participants eligible for the CARS2-HF, the findings may not generalize to populations for whom the CARS2-ST would be more appropriate—such as children under 6 years of age or those with significant communication impairments.

Second, constraints arose from the CARS2 itself. As a clinician-rated observational scale, it integrates caregiver reports, records, and direct observation, and this semistructured flexibility invites variation: one examiner may emphasize caregiver input, while another may emphasize what is seen in the session. In the telehealth arm, parents also acted as camera operators and facilitators, creating an interaction that differed from the clinic, where the clinician directed the child. As the CARS2 lacks a fixed task set, observational opportunities can vary from session to session.

Third, our ICC represented the agreement between two raters working in two different modes; it merged interrater variability with modality effects. We could not fully disentangle the extent of the discrepancy stemming from the telehealth medium (camera angle, bandwidth, and home setting) from ordinary rater subjectivity. Designs using the same clinician in both modes or dual observers watching a single session would better separate these factors but were beyond the scope of this study.

Fourth, home environments introduce uncontrollable noise, such as living rooms or shared spaces with nearby siblings, background media, and limited camera placement. Even with advanced guidance, real-world settings cannot match the controls of a clinical observation room.

Fifth, the assessment interval (≥2 weeks, up to 3 months) may have attenuated agreement relative to a shorter window; future studies with tighter scheduling could test whether ICCs improve while balancing participant burden.

Sixth, the subgroup analyses were exploratory. In particular, the number of girls was small, and several subgroup estimates had wide CIs. We did not conduct formal statistical tests comparing ICCs between diagnostic, sex, or age subgroups. Therefore, apparent differences between subgroup point estimates should be interpreted cautiously.

Taken together, the selected clinical sample, the flexible and partly subjective nature of CARS2 scoring, the combination of rater and assessment-modality effects, the interval between assessments, and variation in home assessment environments limit the generalizability and interpretation of the observed ICCs.

### Conclusions

CARS2 total scores obtained through telepsychiatry showed moderate agreement with scores obtained through face-to-face assessment when the assessments were conducted by different evaluators in different settings. These findings suggest that telepsychiatry-based CARS2 assessment may provide information that overlaps with face-to-face assessment. However, this study does not establish equivalence or interchangeability between the 2 approaches.

Remote CARS2 assessment may have a role as a complementary option when face-to-face assessment is difficult, but comprehensive in person evaluation remains important. Greater standardization of camera positioning, caregiver facilitation, and activities used to elicit relevant behaviors may improve consistency, although these approaches require prospective evaluation. Larger studies involving more diverse populations and designs that separately evaluate rater and modality effects are needed before remote CARS2 assessment can be recommended for routine diagnostic or longitudinal monitoring decisions.

## Supplementary material

10.2196/82784 Checklist 1 CONSORT-EHEALTH (version 1.6.1)

## Abbreviations

ADHD: attention-deficit/hyperactivity disorder
ADHD-RS-IV: Attention-Deficit/Hyperactivity Disorder Rating Scale-IV
ADI-R: Autism Diagnostic Interview-Revised
ADOS: Autism Diagnostic Observation Schedule
ADOS-2: Autism Diagnostic Observation Schedule, Second Edition
ASD: autism spectrum disorder
CARS2: Childhood Autism Rating Scale, Second Edition
CARS2-HF: Childhood Autism Rating Scale, Second Edition—High-Functioning Version
CARS2-ST: Childhood Autism Rating Scale, Second Edition—Standard Version
*DSM-5*: *Diagnostic and Statistical Manual of Mental Disorders (Fifth Edition)*
ICC: intraclass correlation coefficient
rCARS2: remote Childhood Autism Rating Scale, Second Edition
